# Liver failure following biliopancreatic diversions: a narrative review: Falência hepática após derivações biliopancreáticas: uma revisão narrativa

**DOI:** 10.1590/1516-3180.2016.0129220616

**Published:** 2016-11-10

**Authors:** Everton Cazzo, José Carlos Pareja, Elinton Adami Chaim

**Affiliations:** I MD, MSc, PhD. Assistant Lecturer, Department of Surgery, Universidade Estadual de Campinas (Unicamp), Campinas (SP), Brazil.; II MD, PhD. Associate Professor, Department of Surgery, Universidade Estadual de Campinas (Unicamp), Campinas (SP), Brazil.; III MD, MSc, PhD. Full Professor, Department of Surgery, Universidade Estadual de Campinas (Unicamp), Campinas (SP), Brazil.

**Keywords:** Liver failure, Biliopancreatic diversion, Bariatric surgery, Obesity, Liver diseases

## Abstract

**CONTEXT AND OBJECTIVE:**

Occurrences of liver failure following jejunoileal bypass were extensively reported in the past and were one of the main factors that led to abandonment of this procedure. The newer predominantly malabsorptive procedures called biliopancreatic diversions (BPDs) have also been implicated in several cases of acute and subacute liver failure. The aim here was to review the current available evidence on occurrences of liver failure following BPDs.

**DESIGN AND SETTING:**

Narrative review; bariatric surgery service of a public university hospital.

**METHODS:**

A review of the literature was conducted through an online search of medical databases.

**RESULTS:**

Associations between BPDs and liver failure have only infrequently been reported in the literature. However, they appear to be more than merely anecdotal. The pathophysiological mechanisms remain obscure, but they seem to be related to rapid weight loss, protein malnutrition, deficits of hepatotrophic factors, high circulating levels of free fatty acids and bacterial overgrowth in the bypassed bowel segments. Reversal of the BPD may ameliorate the liver impairment.

**CONCLUSIONS:**

Although infrequent, liver failure remains a concern following BPDs. Careful follow-up is required in individuals who undergo any BPD.

## RESUMO

**CONTEXTO E OBJETIVO:**

A ocorrência de falência hepática após a derivação jejunoileal foi extensivamente descrita no passado e foi um dos principais fatores que levaram ao abandono do procedimento. Os procedimentos predominantemente malabsortivos mais modernos, chamados de derivações biliopancreáticas, também já foram implicados em diversos casos de falência hepática aguda e subaguda. O objetivo foi revisar a atual evidência disponível sobre a ocorrência de insuficiência hepática após derivações biliopancreáticas.

**TIPO DE ESTUDO E LOCAL:**

Revisão narrativa; Serviço de Cirurgia Bariátrica de hospital universitário.

**MÉTODOS:**

Revisão da literatura conduzida por meio de pesquisa *online* de bancos de dados médicos.

**RESULTADOS:**

A associação entre derivações biliopancreáticas e falência hepática na literatura é infrequente. Entretanto, ela aparenta ser mais do que meramente anedótica. Os mecanismos fisiopatológicos continuam pouco compreendidos, mas parecem estar relacionados à rápida perda de peso, desnutrição proteica e déficit de fatores hepatotróficos, altos níveis circulantes de ácidos graxos livres e supercrescimento bacteriano em segmentos intestinais excluídos do trânsito. A reversão da cirurgia pode melhorar o comprometimento hepático.

**CONCLUSÕES:**

Embora infrequente, a falência hepática continua sendo preocupante após as derivações biliopancreáticas. Seguimento cuidadoso é mandatório em indivíduos submetidos a essas cirurgias.

## INTRODUCTION

Obesity has become a worldwide public health concern over recent decades. In 2014, according to the World Health Organization (WHO), more than 1.9 billion adults were at least overweight; of these, over 600 million were obese.^1^ Recent reports have observed that the prevalence of obesity in Brazil is 17.9%, which corresponds to almost thirty million obese people.^2^ Bariatric surgery has become the standard treatment for refractory morbid obesity nowadays. Brazil is currently the country with the second largest number of bariatric surgery procedures performed every year, only behind the United States.^3^

The first bariatric procedures, which were described as early as in the 1950s, were jejunocolic and jejunoileal bypasses. Jejunoileal bypass was characterized as a bypass from the proximal jejunum to the distal ileum, with exclusion of the majority of the small bowel of the digestive tract. Despite its popularity from the 1950s to the 1970s, it was abandoned especially because of high rates of severe protein-calorie malnutrition and acute and subacute liver failure related to the procedure. However, the newer predominantly malabsorptive procedures called biliopancreatic diversions (BPDs) have also been implicated in several cases of acute and subacute liver failure.^4^

BPD mainly encompasses two different bariatric procedures: the Scopinaro operation and the duodenal switch procedure. The Scopinaro operation basically involves distal gastrectomy with a bypass from the remnant stomach to the distal ileum. The duodenal switch consists of resection of the gastric greater curvature and distal bypass from the duodenum to the ileum. The duodenal switch procedure is a modification of the original BPD and uses a longer common channel than the classic BPD. It was designed to improve gastric emptying and to decrease postoperative diarrhea and anastomotic ulceration. Both procedures are associated with high rates of resolution of type 2 diabetes. However, they are also associated with occurrences of protein-calorie malnutrition. Hence, although they are still performed today, they are not routinely the operations of choice in most centers.^5^ In the 2003 IFSO report, the Scopinaro operation accounted for 2% and duodenal switch for 2.8%.^6^ In the latest report (in 2013) from the International Federation of Surgery of Obesity and Metabolic Disorders (IFSO), duodenal switch accounted for about 1.5% of all bariatric procedures performed throughout the world, while the statistics for the Scopinaro operation did not appear, since it accounted for less than 1%.^7^

Regarding liver disease, BPDs are usually linked to major improvements in metabolic abnormalities relating to nonalcoholic fatty liver disease (NAFLD), especially insulin resistance, but at the same time, there has been a steady rate of occurrence of reports of acute and subacute liver failure following BPDs over the years.

## OBJECTIVES

This study aimed to review the current available evidence on occurrences of liver failure following biliopancreatic diversions.

## METHODS

A review of the literature was conducted through an online search for the MeSH terms "liver failure", "biliopancreatic diversion" and "bariatric surgery" in Medline (via PubMed); and the MeSH/DeCS terms "liver failure", "biliopancreatic diversion" and "bariatric surgery" in Lilacs (via Bireme) (Table 1).

**Table 1:** Database search results for liver failure following biliopancreatic diversions, on May 22, 2016

Electronic databases	Search strategies	Results
MEDLINE (PubMed)	(Liver failure) AND (Biliopancreatic Diversion) AND (Bariatric surgery)	3 case series 4 case reports
LILACS (Bireme)	((Liver failure) OR (Fallo hepático) OR (Falência Hepática)) AND ((Biliopancreatic Diversion) OR (Desviación Biliopancreática) OR (Desvio biliopancreático) AND ((Bariatric surgery) OR (Cirurgia Bariátrica) OR (Cirurgia bariátrica))	2 case series 3 case reports

After extensive online research, we identified three case reports and two case series on liver failure subsequent to the classical Scopinaro operation; and one case report and one case series of liver failure subsequent to the duodenal switch procedure. Additionally, we also researched population studies that addressed the evolution of liver disease after biliopancreatic diversions and identified two large cohort studies (one retrospective and other prospective) on liver impairment subsequent to the classical Scopinaro operation; and two retrospective cohorts on liver impairment subsequent to duodenal switch. We also excluded two case reports on liver failure after jejunoileal bypass: one case report on liver failure after biliointestinal bypass and one case series on liver failure after conversion of classical gastric bypass to distal bypass.

## RESULTS

### Scopinaro operation

The reports on liver failure requiring liver transplantation or leading to death following the Scopinaro operation are more than anecdotal. Although the rate of occurrence of liver failure appears to be non-significant in large cohort studies, there is enough evidence to consider that these occurrences in individuals who underwent this procedure are more than mere coincidence.^8^

Grimm et al.^9^ reported the first case of chronic end-stage cirrhosis after BPD in 1992. The first successful liver transplantation to treat this complication was reported by Castillo et al. in 2001.^10^ Greco et al.^11^ reported the case of an individual who developed liver failure 16 years after undergoing the Scopinaro operation and presented partial recovery of liver function after the primary procedure had been dismantled. D'Albuquerque et al.^12^ reported on three cases of liver failure that occurred between seven and 24 months after the Scopinaro operation: two of the patients underwent liver transplantation and one died. In a survey on liver transplantation centers in Belgium, Geerts et al.^8^ detected 10 cases of bariatric surgery-related liver failure, of which nine were caused by the Scopinaro operation and one by jejunoileal bypass; the median time taken to develop liver failure was five years. All of these authors emphasized that, along with transplantation, the intestinal bypass must be revised and the original procedure must be dismantled.^8^ ^,^ ^10^ ^,^ ^11^ Table 2 summarizes the main articles on liver failure subsequent to the Scopinaro operation.

**Table 2:** Articles on liver failure subsequent to the Scopinaro operation

Author	n	Treatment	Outcome
Grimm et al.^9^	1	Supportive therapy	Death
Castillo et al.^10^	1	Liver transplantation	Successful
Greco et al.^11^	1	Reversal of intestinal bypass	Successful
D'Albuquerque et al.^12^	3	2: Liver transplantation 1: Supportive therapy	2: Successful (liver transplantation) 1: Death awaiting a graft
Geerts et al.^8^	10 (9: BPD; 1: jejunoileal bypass)	7: Liver transplantation 2: Supportive therapy 1: Awaiting transplantation	4: Successful 1: Successful transplantation followed by death due to *"de novo"* cancer 6 years later 2: Death after transplantation 1: Jejunoileal bypass - reappearance of liver failure 10 months after transplantation; required retransplantation 2: Death while awaiting graft

Despite the reports of liver failure, large population studies have not identified a significant frequency of occurrence of this complication. Scopinaro et al. conducted a classical retrospective analysis on 2,241 individuals who underwent their procedure and did not identify a single case of liver failure.^15^ Papadia et al. did not find any cases of liver failure in a prospective study that enrolled 99 consecutive subjects who underwent the same procedure. However, they observed significant early transient hepatocellular necrosis following the procedure, and noted that individuals with abnormalities seen previously through liver histological analysis were more likely to present postoperative acute liver damage.^16^ Table 3 summarizes the main findings from these population studies.

**Table 3:** Population-based studies evaluating liver impairment following Scopinaro operation and duodenal switch

Study	Surgical technique	n	Study design	Cases of liver failure - n (%)
Scopinaro et al.^15^	Scopinaro	2,241	Retrospective cohort	0
Papadia et al.^16^	Scopinaro	99	Prospective cohort	0
Baltasar et al.^18^	Duodenal switch	470	Retrospective cohort	1 (0.2%)
Keshishian et al.^19^	Duodenal switch	697	Retrospective cohort	0

### Duodenal switch

Although the duodenal switch procedure has been more frequently performed than the Scopinaro operation, at least since the 2000s,^6^ liver failure appears to be less frequent with this technique than with the classical Scopinaro operation. However, some cases have been reported. Auclair et al.^13^ reported the first case of liver failure following duodenal switch, which underwent successful liver transplantation. Baltasar^17^ and Baltasar et al.^18^ reported on two cases of liver failure following duodenal switch, of whom one underwent transplantation and the other died while on the waiting list. Table 4 summarizes the main articles on liver failure subsequent to duodenal switch.

**Table 4:** Articles on liver failure subsequent to duodenal switch

Author	n	Treatment	Outcome
Auclair et al.^13^	1	Liver transplantation	Successful
Baltasar^17^	2	1: Liver transplantation 1: Supportive therapy (while awaiting graft)	1: Liver transplantation - successful 1: Died while awaiting graft

The exact mechanisms that lead to liver failure following BPD and its current prevalence remain uncertain. Baltasar et al.^18^ observed, in a large population study that enrolled 470 individuals who underwent duodenal switch, that only 10 (2.1%) of them developed liver impairment, ranging from asymptomatic liver enzyme abnormalities to fatal acute liver failure. Conversely, in a study that enrolled 697 individuals, Keshishian et al.^19^ found no evidence of liver impairment following duodenal switch. The main findings of these population-based studies are summarized in Table 3.

### Pathophysiology

The pathophysiological pathways potentially enrolled in development of liver failure following BPD appear to consist of early rapid weight loss, a degree of protein malnutrition, lack of hepatotrophic factors and the effect of high levels of mobilized circulating free fatty acids.^17^ ^,^ ^18^ ^,^ ^19^ Exclusion of the long jejunoileal loop can lead to injury to the intestinal mucosal barrier due to nonuse or to functional exclusion of the alimentary bolus. The resulting impaired function of the mucosal barrier may facilitate absorption into the portal venous system of a variety of macromolecules, such as inflammatory cytokines and intestinal toxins arising as a result of changes to the intestinal bacterial flora. After delivery to the liver, these macromolecules may exacerbate hepatic injury.^12^

Even in individuals who do not develop liver failure, BPDs seem to promote a bimodal effect in liver function tests, with early worsening of liver injury, followed by normalization and improvement.^9^ ^,^ ^17^ The reversal of some hepatic features following dismantling of the gut bypass emphasizes the role that this procedure plays in relation to the onset of liver failure. It is possible to propose that the procedure may trigger this change in individuals who are somewhat predisposed towards this. The predisposition factors involved are yet to be identified. In any case, it is reasonable to consider that this surgery is unjustifiable for obese individuals who currently present signs of fibrosis, steatohepatitis and advanced liver disease. Moreover, all individuals undergoing BPD should be carefully followed up, at least by means of serial liver enzyme tests, not just in the early postoperative period, but for their entire lifetime.^8^

## CONCLUSIONS

Although very rare, liver failure remains a concern following BPDs. However, since the vast majority of the evidence available is from case reports, there is no evidence level sufficient to provide definite conclusions. Randomized trials comparing the different available bariatric techniques are needed in order to provide data of better quality. Nonetheless, despite the low frequency of occurrences of liver failure, such events are reported nowhere near as often following other, more frequently performed bariatric techniques. The exact mechanism that leads to this ominous complication remains to be determined, but it seems to be characterized by an acute-on-chronic failure that occurs in predisposed individuals who present previous liver impairment. Careful follow-up is required among individuals who undergo any BPD. Reversal of the procedure is warranted when early clinical or laboratory signs of liver failure appear. Despite the lack of specific evidence, it is reasonable to avoid this surgical technique among subjects who present to bariatric surgeons with any degree of significant liver function impairment.
